# COVID-19 Vaccine-Related Thrombosis: A Systematic Review and Exploratory Analysis

**DOI:** 10.3389/fimmu.2021.729251

**Published:** 2021-11-29

**Authors:** Clio Bilotta, Giulio Perrone, Valeria Adelfio, Giovanni Francesco Spatola, Maria Laura Uzzo, Antonina Argo, Stefania Zerbo

**Affiliations:** ^1^ Department of Health Promotion, Mother and Child Care, Internal Medicine and Medical Specialties, Section of Legal Medicine, University of Palermo, Palermo, Italy; ^2^ Department of Economics, Business and Statistics, University of Palermo, Palermo, Italy; ^3^ Department of Biomedicine, Neurosciences and Advanced Diagnostics (BiND), University of Palermo, Palermo, Italy

**Keywords:** COVID-19 vaccine, vaccine-related thrombosis, vaccine adverse effects, vaccine complications, PF4 antibodies

## Abstract

**Introduction:**

The World Health Organization declared the coronavirus disease 2019 (COVID-19) pandemic on March 11, 2020. Two vaccine types were developed using two different technologies: viral vectors and mRNA. Thrombosis is one of the most severe and atypical adverse effects of vaccines. This study aimed to analyze published cases of thrombosis after COVID-19 vaccinations to identify patients’ features, potential pathophysiological mechanisms, timing of appearance of the adverse events, and other critical issues.

**Materials and Methods:**

We performed a systematic electronic search of scientific articles regarding COVID-19 vaccine-related thrombosis and its complications on the PubMed (MEDLINE) database and through manual searches. We selected 10 out of 50 articles from February 1 to May 5, 2021 and performed a descriptive analysis of the adverse events caused by the mRNA-based Pfizer and Moderna vaccines and the adenovirus-based AstraZeneca vaccine.

**Results:**

In the articles on the Pfizer and Moderna vaccines, the sample consisted of three male patients with age heterogeneity. The time from vaccination to admission was ≤3 days in all cases; all patients presented signs of petechiae/purpura at admission, with a low platelet count. In the studies on the AstraZeneca vaccine, the sample consisted of 58 individuals with a high age heterogeneity and a high female prevalence. Symptoms appeared around the ninth day, and headache was the most common symptom. The platelet count was below the lower limit of the normal range. All patients except one were positive for PF4 antibodies. The cerebral venous sinus was the most affected site. Death was the most prevalent outcome in all studies, except for one study in which most of the patients remained alive.

**Discussion:**

Vaccine-induced thrombotic thrombocytopenia (VITT) is an unknown nosological phenomenon secondary to inoculation with the COVID-19 vaccine. Several hypotheses have been formulated regarding its physiopathological mechanism. Recent studies have assumed a mechanism that is assimilable to heparin-induced thrombocytopenia, with protagonist antibodies against the PF4–polyanion complex. Viral DNA has a negative charge and can bind to PF4, causing VITT. New experimental studies have assumed that thrombosis is related to a soluble adenoviral protein spike variant, originating from splicing events, which cause important endothelial inflammatory events, and binding to endothelial cells expressing ACE2.

**Conclusion:**

Further studies are needed to better identify VITT’s pathophysiological mechanisms and genetic, demographic, or clinical predisposition of high-risk patients, to investigate the correlation of VITT with the different vaccine types, and to test the significance of the findings.

## 1 Introduction

The World Health Organization declared the coronavirus disease 2019 (COVID-19) pandemic on March 11, 2020 (1). Severe acute respiratory syndrome coronavirus 2 (SARS-CoV-2), the pathogen causing this infection, has a mortality rate of 2.3% (China) to 7.2% (Italy) and high transmissibility (2, 3). Globally, as of June 15, 2021, there have been 176,156,662 confirmed cases, and among them, 3,815,486 deaths have been reported (4). SARS-CoV-2’s viral characteristics together with globalization of the infections have considerably reduced the possibility of infection containment despite various preventive measures implemented by different governments worldwide. The acceleration of COVID-19 vaccine development has become necessary due to this global state of emergency (5). A few vaccines have been developed and licensed for clinical use in <1 year.

Two such vaccine types were developed using two different technologies: viral vectors, herein adenovirus, and mRNA. Both vaccine types aim to induce an immune response and generation of neutralizing antibodies against the SARS-CoV-2 spike protein. The spike protein, expressed on the virus surface, enables the virus to bind to human target cells and upon entry to reproduce itself. Viral vector vaccines, such as Vaxzevria (AstraZeneca), exploit a weakened version of chimpanzee adenoviruses rendered able to enter and reproduce itself within human cells. The adenovirus codes for the SARS-CoV-2 spike protein in the nucleus, which, upon translation in the cytoplasm and expression, triggers an immune response (6, 7). The COVID-19 mRNA vaccines, such as Comirnaty (Pfizer) and mRNA-1273 (Moderna), contain mRNA molecules encoding spike proteins within human cells (8, 9).

As of June 15, 2021, a total of 2,310,082,345 individuals have been vaccinated (4). However, little is known regarding these experimental vaccines, whose phase IV clinical trial is monitored during the current mass vaccination and will be necessary for identifying, cataloging, and quantifying adverse reactions, since the large sample size would compensate for the reduction in the development and licensing of the vaccine (10).

Different types of vaccine adverse effects have been described, such as anaphylaxis, fever, joint and muscular pain, headache, weakness, nausea, chills, erythema, lymphadenopathies, paresthesia, vomiting, dizziness, arrhythmias, changes in blood pressure, and facial paralysis (11). Thrombosis is one of the most severe and nontypical adverse effects. Some authors have hypothesized an incidence of approximately one in 100,000/1,000,000 individuals who received the adenoviral vector vaccine (12).

Our study aimed to analyze literature reports of thrombosis after COVID-19 vaccine administration to identify patients’ features, potential pathophysiological mechanism, timing of adverse events, and critical issues to prevent further deaths adding to those that are COVID-19 related.

## 2 Methods

**Figure 1:**
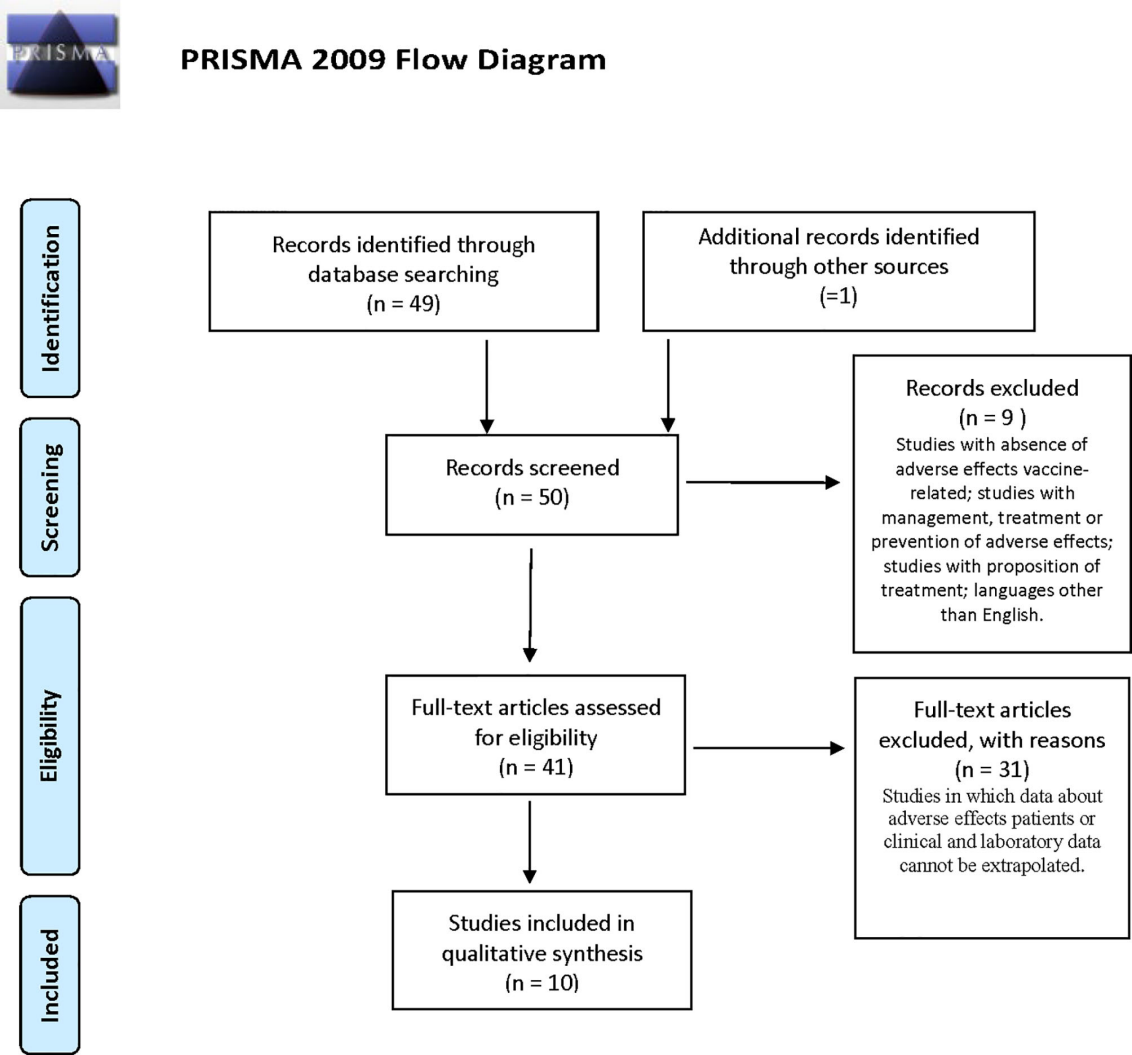
PRISMA flow diagram.

The reviewers performed a systematic electronic search of scientific articles on COVID-19 vaccine-related thromboses and their complications on the PubMed (MEDLINE) database and through manual searches. The obtained results were filtered according to the following criteria: English language and human species. We used the following combinations of keywords: “Covid vaccine AND PF4” OR “Covid vaccine AND immune thrombocytopenia”. We found a total of 49 results from PubMed and one through manual searches. We initially selected 41 of the 50 articles, analyzing the titles and abstracts, and, where useful, opening and studying articles in full text. According to the following inclusion criteria, we selected 10 articles from February 1 to May 5, 2021 (**Figure 1**): titles and abstracts related to COVID-19 vaccination and its adverse reactions; full-text articles concerning COVID-19-vaccine-related thrombosis and subsequent hospitalization; and articles reporting data on platelet count and anti-PF4 antibodies of affected patients. The exclusion criteria were articles with absence of data or information on vaccine-related adverse effects, management, laboratory tests or treatment, and languages other than English.

We analyzed 10 articles (13–22) reporting an overall sample of 61 individuals vaccinated with the AstraZeneca, Pfizer, and Moderna vaccines (**Table 1**). The mRNA COVID-19 vaccines were administered in a few cases: Pfizer in only one individual (19) and Moderna in two individuals (16, 17). Hence, we performed only a descriptive analysis of the Pfizer and Moderna vaccines. Meanwhile, an exploratory analysis of the AstraZeneca vaccine was performed because of the small sample size to obtain an unbiased statistical analysis.

**Table 1:** Analyzed studies.

Article	Sample size	Vaccine type	Age/age range (years)	Female(%)	Mean platelet count	PF4 positivity (%)	Major intracranial hemorrhage (%)	Cerebral venous sinus thrombosis (%)	Headaches at admission (%)	Heparin treatment (%)	Platelet transfusion (%)	Death(%)	Time/mean time from vaccination to admission (days)	Onset of symptoms from vaccination (days/mean days)
Greinacher et al. (13)	11	Astrazeneca	22–49	18%	35,300.0	100%	9%	82%	–	36%	0%	55%	–	9.27
Scull y et al. (14)	23	Moderna	21–77	61%	45,227.3	93%	17%	57%	–	–	–	30%	12.43	–
Schultz et al (15)	5	Moderna	32–54	80%	27,000.0	100%	–	80%	80%	20%	100%	60%	8.40	–
Helms et al. (16)	1	Astrazeneca	74	0%	–	–	–	–	0%	0%	100%	0%	1	1
Mal ayal a et al. (17)	1	Pfizer	60	0%	–	–	–	–	0%	–	–	0%	2	2
Bj ørnsta d-Tuveng et al. (18)	1	Astrazeneca	30–39	100%	37,000.0	100%	100%	100%	83%	0%	0%	100%	7.00	7-00
Lee et al. (19)	1	Astrazeneca	22	0%	–	–	–	–	0%	0%	100%	0%	3	3
See et al. (20)	12	Astrazeneca	18–40	–	45,750.0	100%	–	100%	–	–	–	25%	16.06	8.83
Blauenfeldt et al. (21)	1		60	100%	5,000.0	100%	–	–	–	0%	100%	100%	7.00	1.00
Tiede et al. (22)	5		41–67	100%	49,200.0	100%	–	20%	80%	20%	40%	–	8.40	–

## 3 Results

### 3.1 Pfizer and Moderna

The sample reported in the three studies (16, 17, 19) comprised of three men (one patient in each study) with ages 74, 60, and 22 years (median age, 52 years). The time from vaccination to admission was ≤3 days in all cases. The total sample of all three studies presented signs of petechiae/purpura at admission. Of the sample, 67% was under antihypertensive therapy. One patient had a clinical history of hepatitis C, chronic heart disease, kidney failure, and immunosuppressive therapy, presenting with a wide range of signs and symptoms, such as chest and abdominal pain, hypertension, dyspnea, nausea, vomiting, and pitting edema.

All three individuals had a platelet count below the lower limit of the normal range (10,000, 84,000, and 2,000) with a median value of 32,000. Only one study (19) specified data on the positivity of platelet antibodies IIb/IIIa and Ia/IIa. Two (16, 19) of the three individuals received corticosteroid therapy, intravenous immunoglobulin (IVIG), and platelet transfusion; one received a thrombopoietin/thrombopoietin receptor agonist (16); and one did not receive treatment (17). No studies described radiological and autopsy findings or thrombosis locations. No deaths were recorded.

### 3.2 AstraZeneca

We analyzed 58 individuals reported in seven studies (13–15, 18, 20–22).

#### 3.2.1 Demographic Characteristics

We explored the demographic characteristics of the sample, although there was a clear limitation in the heterogeneity of the data collection. We decided to report only age range and sex frequencies (**Table 2**).

**Table 2:** Patients demographic.

	Range Age	Male	Female	GenderNA	Size
Blauenfeldt et al. (21)	60	0	1	0	1
Greinacher A. et al. (13)	22–49	9	2	0	11
Bjørnstad-Tuveng et al. (18)	30–39	0	1	0	1
Schultz et al. (15)	32–54	1	4	0	5
Scully et al. (14)	21–77	9	14	0	23
See et al. (20)	18–40+		12	12	
Tiede et al. (22)	41–67	0	5	0	5

The age range is highly heterogeneous and does not allow for the determination of the global range of the sample. The sample showed a high prevalence of the female sex.

#### 3.2.2 Medical History

**Figure 2:**
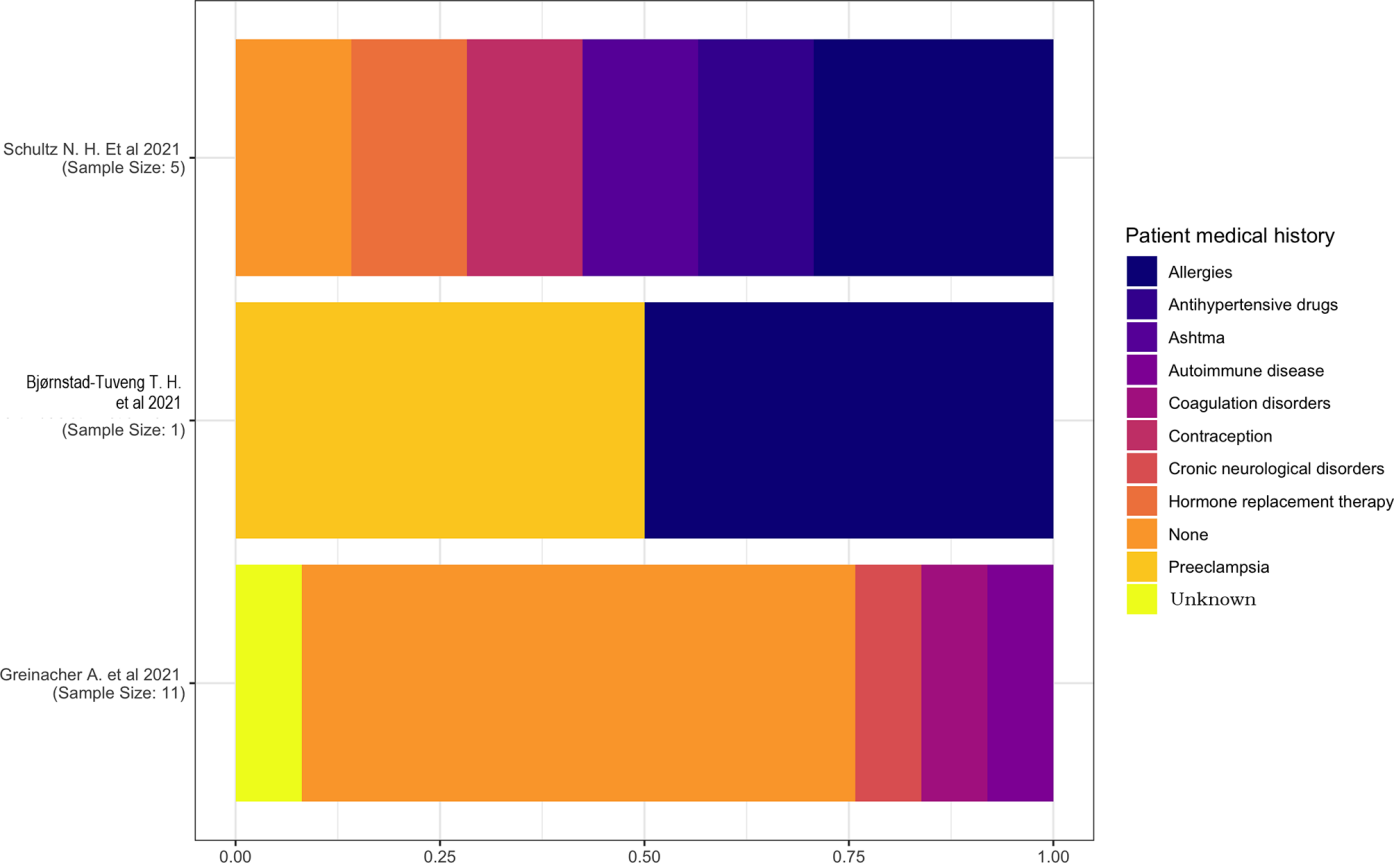
Patient medical history.

The medical history of the patients was reported in only three studies (13, 15, 18). One study (18) had a sample size of a single individual with a medical history characterized by pre-eclampsia and allergies, and another study (15) has reported the prevalence of allergies and an equal distribution of hypertension, asthma, contraception, and hormone replacement therapy (**Figure 2**).

#### 3.2.3 Temporal Relationship

The analysis of the time from vaccination to admission revealed that one study (13) did not report this datum. The two studies (18, 21), with a sample of a single individual, have reported a time distribution of 7 days, while the other studies have reported a range of 8–9 to 16 days (**Table 3**).

**Table 3:** Time from vaccination.

	Mean	S.E.	Size
Blauenfeldt et al. (21)	7.00		1
Greinacher A. et al. (13)			2
Bjørnstad-Tuveng et al. (18)	7.00		1
Schultz et al. (15)	8.40	0.68	4
Scully et al. (14)	12.43	0.82	14
See et al. (20)	16.08	1.33	12
Tiede et al. (22)	8.40	0.98	5

**Figure 3:**
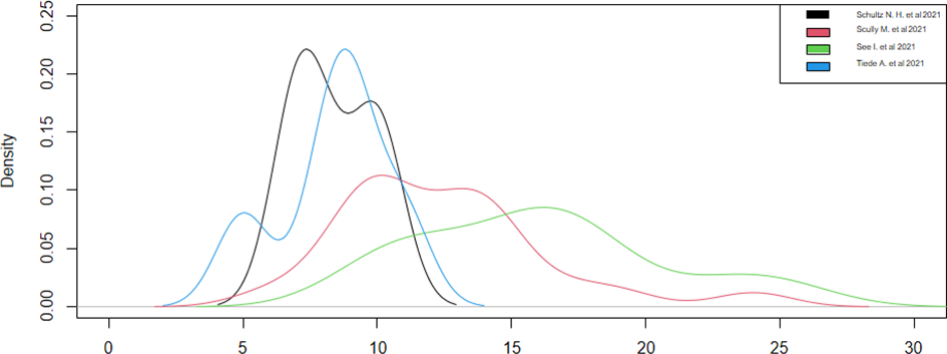
Time from vaccination to admission (days).

We created a graph for the comparison of densities to better analyze the distributions (**Figure 3**), as these studies had different densities. We observed that the patients were admitted on the 15th day after the vaccination in the studies with a larger sample size and on the 10th day after the vaccination in the studies with a smaller sample size.

**Figure 4:**
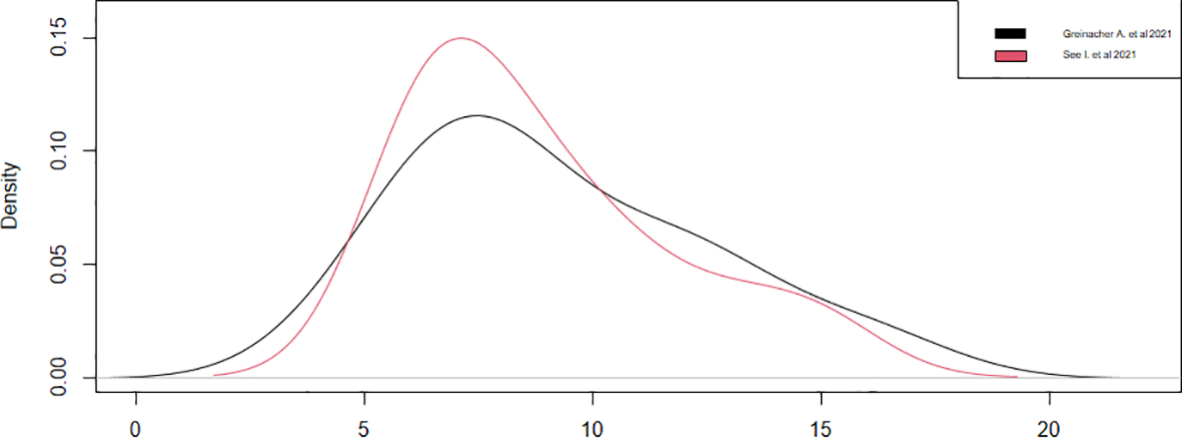
Onset of symptoms from vaccination.

Regarding the onset of symptoms from vaccination, only four studies have reported this datum (13, 18, 20, 21). We could consider only two studies (13, 20), since the other two (18, 21) had a sample of a single case, noticing that the symptoms appeared around the ninth day, with a statistical accuracy of ±1, after the vaccination in both studies (**Table 4**). These results were confirmed by observing the densities (**Figure 4**). Thus, during the first 15 days after vaccination, there is a high risk of developing all adverse symptoms.

**Table 4:** Onset of symptoms from vaccination.

	Mean	S.E.	Size
Blauenfeldt et al. (21)	**7.83**		1
Greinacher A. et al. (13)	9.27	1.01	2
Bjørnstad-Tuveng et al. (18)	7.00		1
Schultz et al. (15)			4
Scully et al. (14)			14
See et al. (20)	8.83	0.84	12
Tiede et al. (22)			5

#### 3.2.4 Signs and Symptoms at Admission

**Figure 5:**
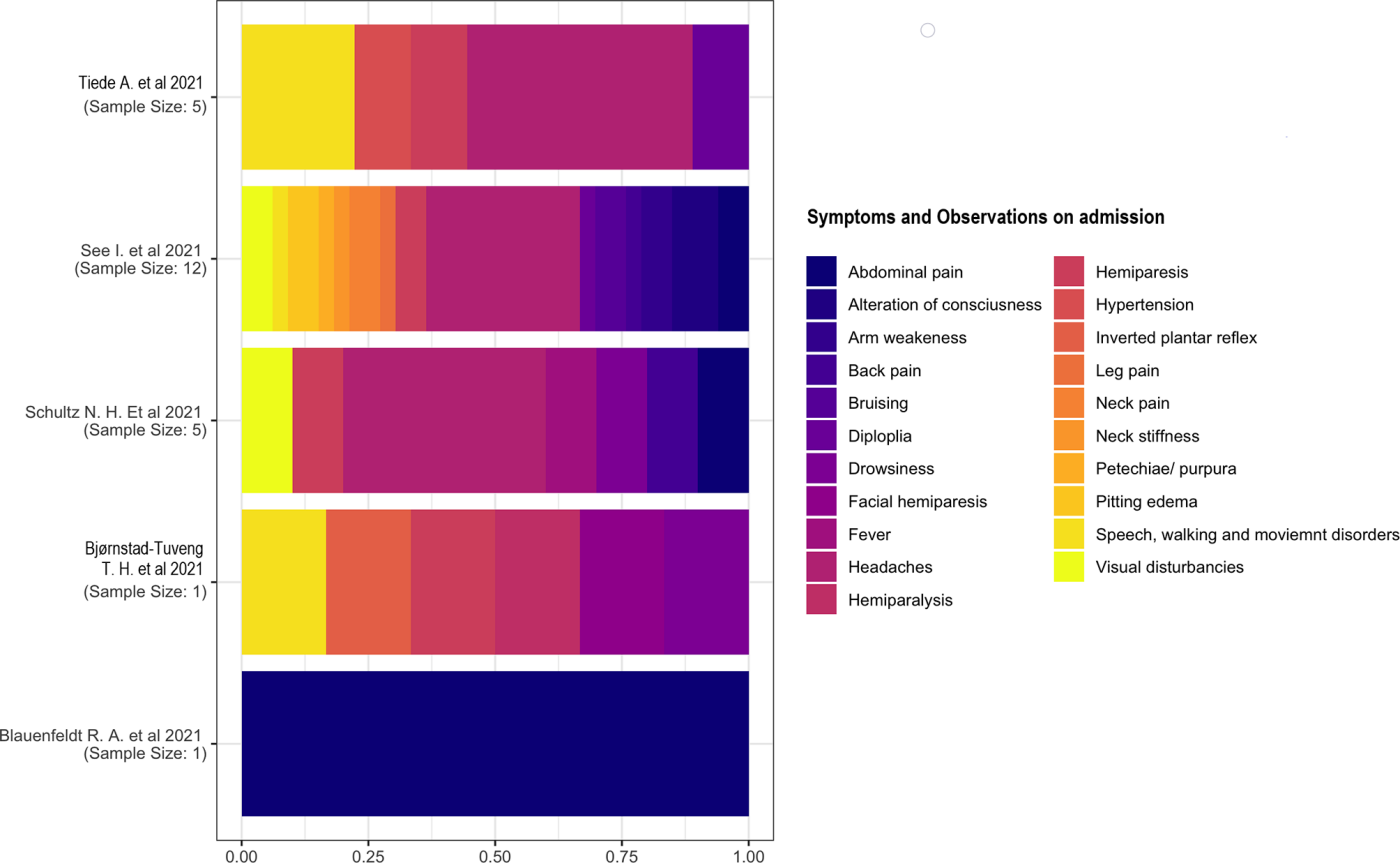
Symptoms and observations on admission.

Five studies (15, 18, 20, 22) have reported signs and symptoms at admission. We considered the percentage based on the total number of symptoms rather than the number of individuals because of the presence of multiple symptomatology in each individual. We described the two studies separately (18, 21) with a single case as the sample. One study (18) reported a multiple symptomatology consisting of inverted plantar reflex; speech, walking, and movement disorders; hemiparesis; facial paresis; and drowsiness. The other study (21) reported only abdominal pain in a patient. Headache was the most common symptom on admission. The other symptoms had similar proportions (**Figure 5**).

#### 3.2.5 Platelet Count, Fibrinogen, and PF4 Antibody Positivity

The platelet count was below the lower limit of the normal range in each study with different accuracies indicated by the standard error (S.E.) expressing the variation in the average estimate. The absence of S.E. indicates a lack of data in the respective study.

The average fibrinogen level was within the normal range (150–400 mg/dl) in all studies (**Table 5**).

**Table 5:** Laboratory data (platelet count and fibrinogen).

	Mean platelet	S.E.P.	Mean fibrinogen	S.E.F.	S.E.F.
Blauenfeldt et al. (21)	5,000.0	374.0			1
Greinacher A. et al. (13)	35,300.0	10,210.9	191.5	58.9	11
Bjørnstad-Tuveng et al. (18)	37,000.0	220.00			1
Schultz et al. (15)	27,000.0	10,945.3	152.0	28.9	5
Scully et al. (14)	45,227.3	6,788.1	187.8	25.0	23
See et al. (20)	45,750.0	12,252.5	159.3	23.6	12
Tiede et al. (22)	49,200.0	16,184.6			5

We studied the prevalence distribution of anti-PF4 antibodies, highlighting the complete positivity (100%) of the sample, except for one patient (93%) (**Table 6**).

**Table 6:** PF-4 antibodies value (ELISA).

	Negative	Positive	NA	Size
Blauenfeldt et al. (21)	0.00	1.00	0	1
Greinacher A. et al. (13)	0.00	1.00	2	11
Bjørnstad-Tuveng et al. (18)	0.00	1.00	0	1
Schultz et al. (15)	0.00	1.00	0	5
Scully et al. (14)	0.00	1.00	8	23
See et al. (20)	0.00	1.00	1	12
Tiede et al. (22)	0.00	1.00	0	5

#### 3.2.6 Autopsy Findings

**Figure 6:**
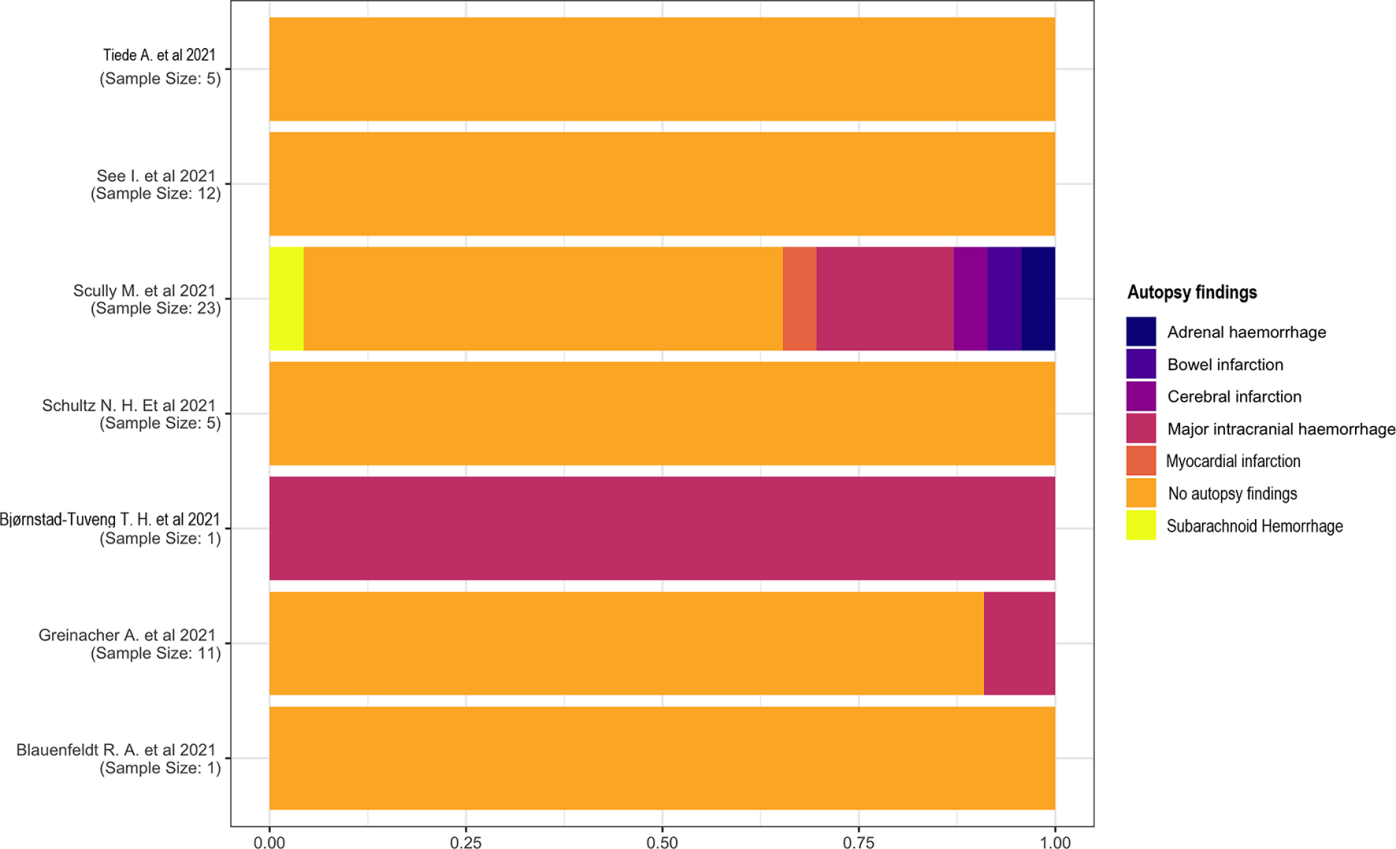
Autopsy findings.

Most studies did not describe autopsy findings. One study (18) reported a major intracranial hemorrhage in one patient. This complication has also been reported in two other studies (13, 14), one of which (14) had a high heterogeneity describing subarachnoid hemorrhage, myocardial infarction, cerebral and bowel infarction, and adrenal hemorrhage (**Figure 6**).

#### 3.2.7 Thrombosis Location

**Figure 7:**
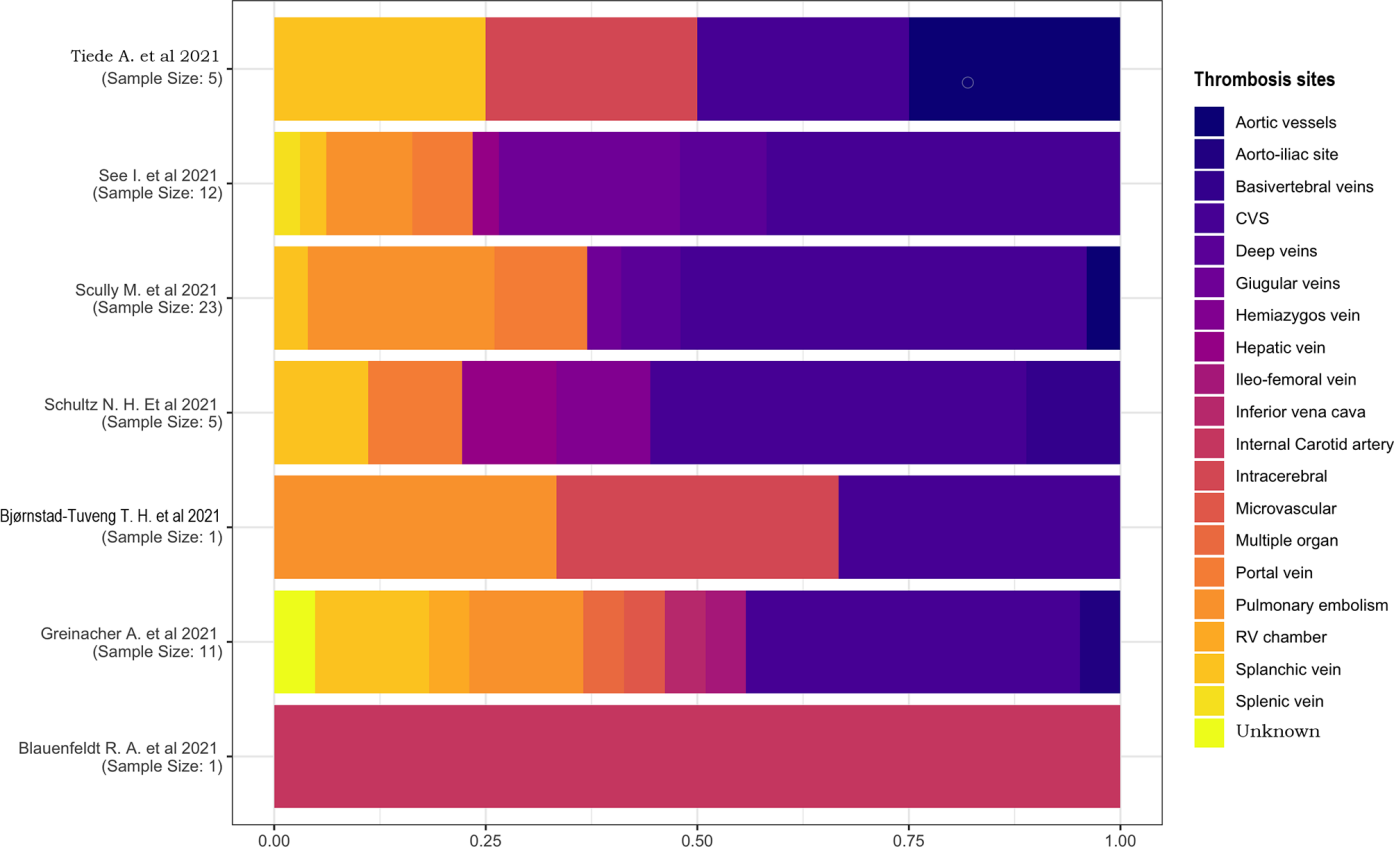
Thrombosis sites.

We examined the proportions of thrombosis location (**Figure 7**) and performed the calculation based on the number of thromboses rather than the number of individuals. Thrombosis could affect more vessels in each patient, so it is possible that the number of occluded blood vessels surpasses the number of patients; the ratio is >1 in this case.

Only two studies had a sample of one patient (18, 21), one of which described a case of internal carotid artery thrombosis, and the other one had a multiple-located thrombosis involving the cerebral venous sinus (CVS), intracerebral, and pulmonary vessels. In the other studies, CVS was the most affected location, with a percentage of 25%–45%. No other proportions were comparable because of their high heterogeneity and small sample sizes.

#### 3.2.8 Treatments

**Figure 8:**
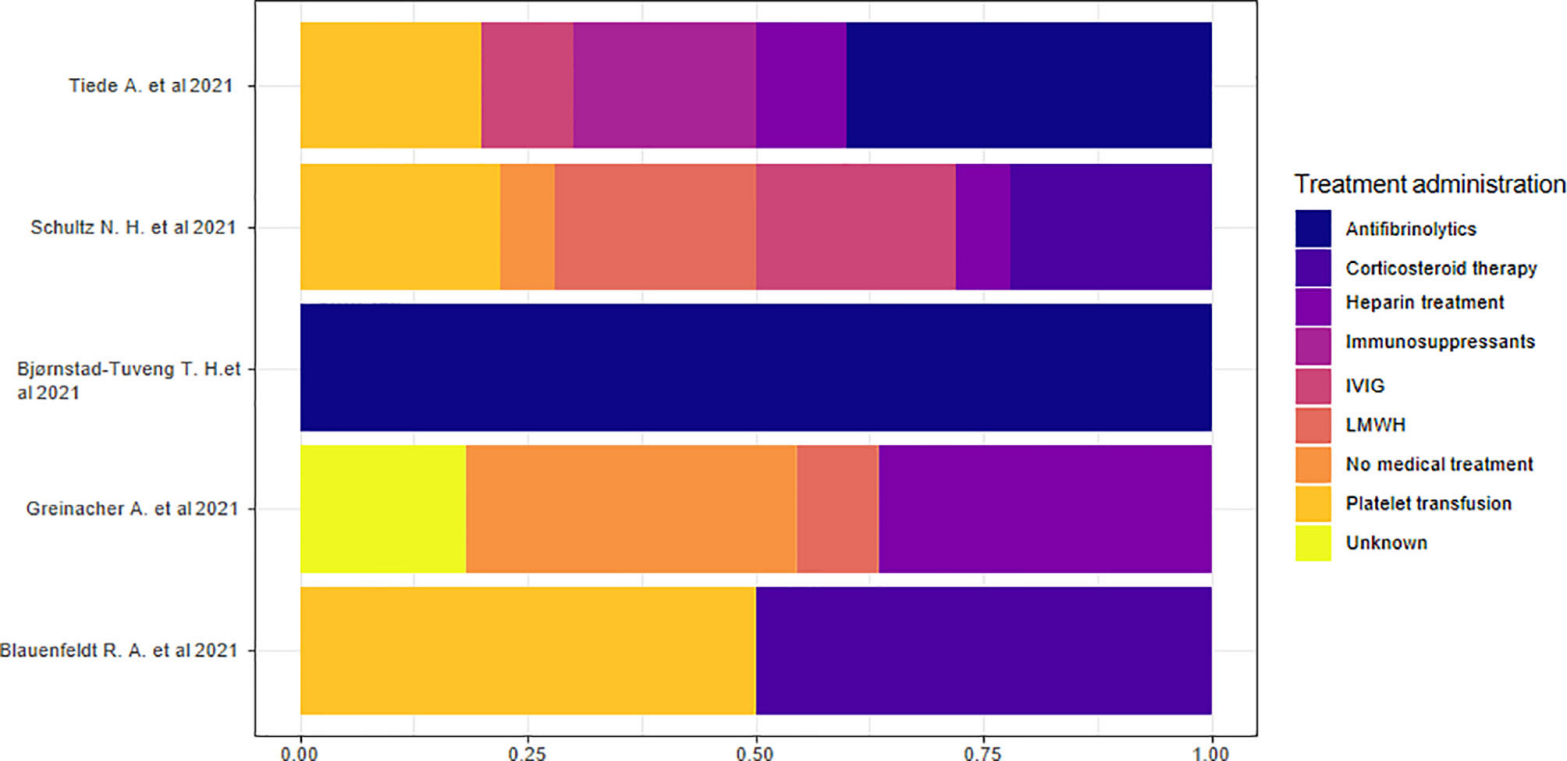
Treatment administration.

Five studies reported data on hospital treatment (13, 15, 18, 21, 22). We considered the percentage based on the total number of treatments rather than the number of individuals because of the presence of multiple therapies in each individual. We highlighted the absence of a specific treatment prevalence in the overall sample. The analysis of the proportions of single studies indicated that antifibrinolytic treatment was the most commonly used therapy in two studies (18, 22) and corticosteroid therapy in two other studies (15, 21). Heparin treatment or no medical treatment was the prevalent therapeutic approach in another study (13) (**Figure 8**).

#### 3.2.9 Outcomes

Five studies (13–15, 18, 21) specified patient outcomes. Both patients died in the cases described in the two studies with a sample size of a single individual. Death was the prevalent outcome in all studies, except for one study (14) with a reported survival rate of 70% (**Table 7**).

**Table 7:** Outcome.

	Alive	Death	Size
Blauenfeldt et al. (21)	0.00	1.00	1
Greinacher A. et al. (13)	0.40	0.60	2
Bjørnstad-Tuveng et al. (18)	0.00	1.00	1
Schultz et al. (15)	0.40	0.60	4
Scully et al. (14)	0.70	0.30	14

## 4 Discussion

The current mass vaccination led to the discovery of vaccine-induced thrombotic thrombocytopenia (VITT), an unknown nosological phenomenon secondary to the inoculation of the COVID-19 vaccine (13–15, 18, 21). The pathophysiological mechanism behind this adverse reaction remains unclear. Recent studies have assumed a mechanism that is similar to heparin-induced thrombocytopenia (HIT) (13). The latter represents a known adverse reaction that remains one of the most serious complications in individuals treated with heparin (23). A platelet count decrease of >50% and a procoagulant state with a secondary development of venous or arterial thrombosis represent the main diagnostic criteria from a clinical laboratory point of view (24).

There may be an immune-mediated mechanism at the root of thrombosis, with protagonist antibodies against the PF4–polyanion complex. VITT-associated PF4 antibodies interact with the heparin-binding site. These antibodies are therefore independent from heparin. The immune complexes, formed from the binding of PF4 to antibodies, activate platelets through FcγRIIa receptors, causing thrombocytopenia and thrombosis secondary to the activation of the coagulation pathway. Therefore, *in vitro*, heparin does not increase the activation of platelets in case of thrombosis with thrombocytopenia syndrome (TTS); heparin could inhibit platelets’ activation secondary to a pharmacological antagonism. The independence from heparin denotes a similarity between TTS and HIT due to heparin-independent antibodies; the main differences consist of the needed previous exposure to heparin in HIT and its greater incidence rate than VITT. The antibodies of HIT heparin independent and TTS have a high affinity for the receptor and are able to bind it in the absence of heparin. Previous studies discovered that HIT heparin-independent antibodies can activate platelets in the presence of adenoviral vector and in a dose-related manner. In fact, HIT is also caused by various mechanisms, which include polymorphism of FcγRIIa receptor (25), monocyte activation, production of tissue factor (26), and generation of procoagulant microparticles (27). These findings suggest that the PF4 antibodies-mediated platelet activation may not be the only cause for thrombosis in VITT (13). In fact, polyanions including nucleic acids and components of the bacterial cell wall, with their negative charge, promote binding to PF4, a positively charged chemokine released by platelets during platelet activation (28). It is plausible that the adenoviral SARS-CoV-2-specific proteins could trigger the immunopathological mechanism in VITT; otherwise, other DNA-based vaccines may also cause thrombosis. Antibody cross-reactivity through molecular mimicry between spike proteins and PF4 epitopes has been hypothesized, albeit not yet proven (29). Regarding nucleic acids, binding with PF4 occurs when their plasma concentration exceeds the normal upper limit of 200 ng/ml (30). The viral DNA has a negative charge and can bind to PF4, acting as an adjuvant because of its phosphate groups that can stimulate Toll-like receptor (TLR)-9 on plasmacytoid dendritic cells with the subsequent production of interferon (IFN) alpha (31). RNA is also negatively charged; therefore, it has not been entirely excluded as a cause of cascade of pathological events. The local concentration of nucleic acids increases due to their release by specific cell structures under particular conditions, such as the breakdown of viral and bacterial pathogens, massive tissue damage, and increased apoptosis (32–34). Several authors have reported a correlation between this pathological event and bacteremia, even when it is subclinical, as in the case of periodontitis (33). Other authors have also recently highlighted the viral capacity to trigger this pathological process (35, 36). Moreover, major surgeries, such as knee replacement, may constitute another risk factor (37–40). New experimental studies on vaccine-related thrombosis have recently been performed, suggesting a new possible mechanism (41). They assumed that thrombosis is related to a soluble adenoviral protein spike variant, originating from splicing events, which cause important endothelial inflammatory events, binding to endothelial cells expressing ACE2. This mechanism may also be related to severe cases of SARS-CoV-2 and pseudovirus infections. This would explain the greater correlation of thrombosis with the adenovirus-based rather than with mRNA-based vaccines.

It is also possible that there is a common physiopathological mechanism for COVID-19 infection and VITT or HIT. Some researchers hypothesized an important role of the innate immune system in causing thrombotic events in COVID-19 patients and in cases of VITT or HIT (42).

Neutrophils, which are cells of the innate immune system, are able to capture microorganisms through neutrophil extracellular traps (NETs), which are essential structures for phagocytosis. Dysregulation of this mechanism is responsible for the procoagulant state and the subsequent alveolar damage and coagulopathy of COVID-19 patients (43).

Inflammation and infection are known to stimulate neutrophil recruitment (44). Excessive activation of neutrophils in COVID-19 patients could therefore trigger the coagulopathy secondary to the fibrin deposition during phagocytosis (45). NETs’ components, such as cell DNA, histones, and tissue factor (III) can activate the coagulation. The histones stimulate activation of platelets and endothelium interacting with TLR2 and TLR4 (46–48); on the other hand, platelets induce NETs’ production through P-selectin (49). The NETs expulsion facilitates adhesion and further activation of platelets leading to a self-perpetuating process. Finally, the deposition of fibrin, secondary to NETs, traps bloodstream cells, such as platelets and red blood cells, providing a basis for blood clot formation (49, 50).

In case of VITT, the certain inflammatory vaccine adjuvants and delivery systems could trigger immune system cells’ recruitment, especially neutrophils, through the activation of NPL3 inflammasome and enhance NET production (42, 51, 52). NETs’ components by binding to PF-4 determine the production of antibodies against PF-4 polyanionic complexes.

Concerning the immunological mechanism, it has been hypothesized as either an innate or humoral-mediated response, through the activation of B and T cells, could take part in the pathophysiological mechanism of thrombosis (53–55).

It is assumed that genetic predisposition is based on HIT. Some authors have shown a higher frequency of T-cell death-associated gene 8 (TDAG8) regarding genetic predisposition (56).

Other researchers identified individuals with the FcγRIIA-131RR genotype as a susceptible population, as they could be more efficiently able to activate a procoagulation response by the activation of platelets and monocytes (57, 58).

Therefore, it has been further suggested that monocytes precede the activation of platelets: FcγRIIA receptor triggers a downstream response involving tyrosine kinase-dependent pathway, with a subsequent release of thrombin and tissue factor, which are both coagulation promoters (59).

To date, a correlation of VITT with a specific medical history remains unknown.

Interestingly, in our study, the overall patient samples consisted of men in the articles on the Pfizer and Moderna vaccines, while the patient samples were predominantly female in the articles on the AstraZeneca vaccine. These findings should also be further analyzed to evaluate their significance in larger sample sizes.

The diagnostic criteria of VITT are the following: time of onset ranging from 4 to 30 days after the AstraZeneca or J&J administration, diagnosis of thrombosis, finding of thrombocytopenia, and PF4 HIT ELISA positivity.

The evaluation of the increase in anti-PF4 antibodies in VITT is performed differently than that of HIT because of the absence of hospitalization and monitoring of laboratory parameters after vaccination. Consequently, the increase in antibodies in VITT can be evaluated indirectly by analyzing the time relationship between vaccination and the onset of symptoms. This temporal range is similar to that of HIT in our study, which is in accordance with the literature (23).

The anti-PF4/heparin antibody titers, in fact, has a characteristic temporal pattern: they appear 4–5 to 10–12 days after heparin administration (60) and disappear within 100 days (61). We noticed that symptoms appeared on the 8th to 10th day after vaccination. We can conclude that during the first 15 days after the vaccination, the vaccinees are at high risk of symptom development, and this period may represent the ideal temporal range in thrombosis prevention. Laboratory data monitoring should therefore preferentially be performed in this temporal range.

Based on our study, the most affected thrombosis site was the cerebral venous system, and this is confirmed by the observations in the literature (23), which also points out frequent involvement of the portal circulation. In fact, the portal circulation and the cerebral venous circulation drain the intestines and nasal sinus, respectively, both sites with a high bacterial presence; it facilitates the passage of bacteria, viral products, or toxins in the veins. The interaction between these elements with PF4 could cause an abnormal immune response with platelet and neutrophil activation, in case of high anti-PF4 antibodies titer (23). According to another recent hypothesis, the major incidence of thrombosis in these venous sites could be related to a non-unidirectional blood flow due to body posture in this vascular district, which does not present a valvular system, with a prolonged exposure to the soluble spike protein and a high risk of binding of this protein to endothelial cells expressing ACE2 (41). These sites are different than those affected in the HIT, including adrenal hemorrhagic necrosis, limb thrombosis, disseminated intravascular coagulation, skin necrosis, deep vein thrombosis, left ventricle thrombosis, and endograft thrombosis (62–69).

Cerebral venous sinus thrombosis (CVST) represents a rare pathological disease occurring secondary to blood clots, which obstruct the blood flow of cerebral vessels or dural sinuses (70).

Women of child-bearing age are the most affected subjects (71, 72). The major risk factors are anticoagulation therapy, brain infections, head trauma, pregnancy, and contraception (73, 74). COVID-19 patients presented relatively low incidence of this complication. Recently, some individuals, especially women aged between 18 and 60, developed CSVT after vaccinations with the AstraZeneca and Johnson and Johnson (Ad26.COV2.S) vaccines. The occurrence of these complications temporarily prohibited administration of the J&J vaccine, in the USA and Germany, and of AstraZeneca vaccine in many European countries (75).

The mRNA vaccines, on the other hand, are not associated with this complication (76). CSVT incidence rate hovers around three to four cases per million before COVID-19 vaccinations (77). The incidence rate of CSVT is 1 in 100,000 after AstraZeneca and 1 in 1,000,000 after J&J vaccinations (78).

Clinical presentation includes headache, generalized or localized, in association with hemiparesis and aphasia. Seizures and encephalopathy appear in a minority of such cases, and intracranial hypertension is responsible for their onset.

Secondary symptoms therefore include dyspnea, limbs’ weakness, petechiae, and lethargy (76).

Randomized controlled trials recommended anticoagulation therapy, except for heparin, in order to avoid the extent of the hemorrhage (77, 78). In fact, heparin and heparin-containing products are not recommended due to the risk of progression of thrombosis and the correlation with HIT for the above-mentioned reasons (13).

Some authors propose the administration of plasma exchange, in order to increase the fibrinogen level, or even the use of Bruton tyrosine kinase (Btk) inhibitors. Btk inhibitors could block downstream pathway of FcγRIIA receptor and prevent monocytes and platelets’ activation and NETs’ formation (79).

Finally, the outcome of VITT depends on the extent of venous, arterial, or microcirculatory thrombotic complications (80, 81). We highlighted a negative outcome in all studies reporting these data, except for the largest study with a prevalent positive outcome.

Specific guidelines could be created on the basis of the event’s frequency and the eventual risk of revealing patient identity, similar to those for HIT, recommending close monitoring of at risk patients, every 2–3 days, especially during the above-mentioned time interval of the first 15 days after vaccination (82, 83).

Further studies are needed to better identify VITT pathophysiological mechanisms, the importance of genetic, demographic, or clinical predisposition, the characteristics of high-risk patients, the correlation of thrombosis with the different vaccine types, and the statistical significance of the findings.

### 4.1 Study Limitations

The small sample size and heterogeneous data collection allowed us to perform an exploratory and descriptive analysis and hence not testing the statistical significance of the findings.

## Data Availability Statement

The original contributions presented in the study are included in the article/supplementary material. Further inquiries can be directed to the corresponding author.

## Author Contributions

CB and GP set the systematic review, selected and analyzed data, and wrote the results and discussion. VA processed the statistical data and wrote the statistical results. SZ and AA reviewed the selected data and wrote a discussion and conclusion. GS and MU analyzed the clinical and histopathological data. All authors contributed to the article and approved the submitted version.

## Conflict of Interest

The authors declare that the research was conducted in the absence of any commercial or financial relationships that could be construed as a potential conflict of interest.

## Publisher’s Note

All claims expressed in this article are solely those of the authors and do not necessarily represent those of their affiliated organizations, or those of the publisher, the editors and the reviewers. Any product that may be evaluated in this article, or claim that may be made by its manufacturer, is not guaranteed or endorsed by the publisher.
